# Supplementary material for: MetaRibo-Seq measures translation in microbiomes
Source: Nat Commun. 2020 Jun 29;11:3268. doi: 10.1038/s41467-020-17081-z (PMC7324362; doi:10.1038/s41467-020-17081-z)
Supplement: Supplementary file 10 — Supplementary Data 7 [file 41467_2020_17081_MOESM10_ESM.zip › File2/Confidence_High_Taxonomy/278037_out.krona.html]

Javascript must be enabled to view this page.

members
magnitude
magnitudeUnassigned
count
unassigned
taxon
rank

278037\_out

19

2
19
superkingdom

1239
19
phylum

class
19
186801

order
19
186802

186803
family
19

841
genus
19

19
species
166486

SRS014923\_contig\_number\_40051SRS014948\_contig\_number\_17517SRS017821\_contig\_number\_9898SRS043001\_contig\_number\_contig-100\_1146.69823SRS050925\_contig\_number\_37772SRS050941\_contig\_number\_15836SRS058070\_contig\_number\_contig-100\_252.188805SRS075341\_contig\_number\_contig-100\_930.150831SRS1055022\_contig\_number\_10004SRS144362\_contig\_number\_23667SRS144537\_contig\_number\_45137SRS144714\_contig\_number\_contig-100\_768.168720SRS147919\_contig\_number\_18569SRS148091\_contig\_number\_14020SRS148159\_contig\_number\_34874SRS148784\_contig\_number\_contig-100\_2032.147656SRS149181\_contig\_number\_contig-100\_1060.175734SRS149879\_contig\_number\_24255SRS893378\_contig\_number\_23483
